# Single-cell high-dimensional imaging mass cytometry: one step beyond in oncology

**DOI:** 10.1007/s00281-022-00978-w

**Published:** 2023-01-04

**Authors:** Yaël Glasson, Laure-Agnès Chépeaux, Anne-Sophie Dumé, Virginie Lafont, Julien Faget, Nathalie Bonnefoy, Henri-Alexandre Michaud

**Affiliations:** 1 IRCM, Univ Montpellier, ICM, Plateforme de Cytométrie Et d’Imagerie de Masse, Inserm Montpellier, France; 2 IRCM, Univ Montpellier, ICM, Inserm Montpellier, France

**Keywords:** Imaging mass cytometry, MIBI-TOF, Hyperion Imaging System, Tumor microenvironment, Cellular network

## Abstract

Solid tumors have a dynamic ecosystem in which malignant and non-malignant (endothelial, stromal, and immune) cell types constantly interact. Importantly, the abundance, localization, and functional orientation of each cell component within the tumor microenvironment vary significantly over time and in response to treatment. Such intratumoral heterogeneity influences the tumor course and its sensitivity to treatments. Recently, high-dimensional imaging mass cytometry (IMC) has been developed to explore the tumor ecosystem at the single-cell level. In the last years, several studies demonstrated that IMC is a powerful tool to decipher the tumor complexity. In this review, we summarize the potential of this technology and how it may be useful for cancer research (from preclinical to clinical studies).

## Introduction

Tumor development and progression are modulated by complex intrinsic and extrinsic biological mechanisms. It is now clear that tumors are complex ecosystems composed of malignant and non-malignant cell types, such as cancer-associated fibroblasts (CAF), endothelial cells, and tumor-infiltrating immune cells that constantly interact by secreting soluble factors or through direct ligand-receptor interactions. These interactions change temporally and spatially, and define the tumor microenvironment (TME) architecture. The tumor ecosystem and architecture can be strongly affected by anti-cancer treatments, and change together with the cancer cell clone diversity and genomic alterations through reciprocal influences [1]. Therefore, one of the main challenges in oncology is to increase our understanding of the spatiotemporal changes in the tumor ecosystem composition and architecture. The exploration of intratumor heterogeneity, by determining the spatial distribution of metabolites, RNAs, and proteins in single cells, will lead to a more precise understanding of the tumor cellular and molecular mechanisms. This will contribute to improve patient stratification and to the identification of novel therapeutic targets.

Comprehensive in situ multiplex technologies have been developed to study the TME, while maintaining the cell spatial information [2]. Sequential immunostaining approaches (i.e., PhenoCycler, formerly known as CODEX [3], from AKOYA, or MACSima [4] from Miltenyi) have allowed increasing the number of biomarkers that can be assessed simultaneously. However, these approaches require multiple slide treatment rounds and acquisition steps that may modify the epitope affinity and damage the tissue architecture. They also require a precise image alignment preprocessing step for analysis. In addition, tissue autofluorescence might limit the sensitivity of protein detection. In the 2010s, high-dimensional imaging mass cytometry (IMC) was developed to overcome these limitations, thus offering a level of tissue analysis never achieved before [5, 6].

IMC combines the high-plex capacity of mass cytometry (CyTOF) with in situ immunohistochemistry (IHC). Currently, IMC can be performed using two systems: the Hyperion Imaging System (HIS) and MIBIscope (formerly known as MIBI-TOF) that are distributed by Standard Biotools and by Ionpath, respectively. These systems allow measuring the expression of more than 40 biomarkers simultaneously in tissue sections using a combination of stable metal isotope-conjugated antibodies or DNA probes. Single-cell data, including marker expression and spatial (*X* and *Y*) coordinates, can be extracted using dedicated algorithms. Thus, IMC is a comprehensive technology suitable for tissue exploration and discovery. By allowing the spatial visualization of several markers simultaneously in tissues, IMC precisely describes the tissue architecture for extracting data on cell quantification, phenotype, localization, and also interaction networks. In this review, we describe IMC principle and its usefulness for oncology by discussing the most recent basic, translational, and clinical research studies.

## Imaging mass cytometry principle

### Use of metal-tagged probes

IMC is based on the use of heavy metal-tagged probes that are quantified by time of flight mass spectrometry (TOF–MS) to eliminate autofluorescence and spectral overlaps and to significantly increase the multiplexing capacity. To date, 42 metals in addition of a cationic nucleic acid intercalator that contains natural-abundance iridium (191Ir and 193Ir) identifying nuclei have been used for imaging mass cytometry allowing to identify as many markers (Table 1). IonPath and Standard Biotools propose a limited number of ready-to-use metal-tagged antibodies validated for imaging applications. To extend the antibody panel, metals and conjugation kits are commercially available for in-house conjugation of antibodies. The limit remains the number of available pure metal isotopes [7]. It is also possible to conjugate an antibody that is already tagged with a fluorescent dye, to identify a region of interest by fluorescence microscopy before performing IMC [8]. However, some antibodies cannot support the reduction/oxidation step during the conjugation procedure and may lose the metal tag or cannot be reconstitute, particularly specific isotypes (i.e., IgM, IgY). Therefore, after conjugation, it is highly recommended to confirm the conjugation and its stability, and to titer the conjugated antibody in a relevant tissue.

**Table 1:**
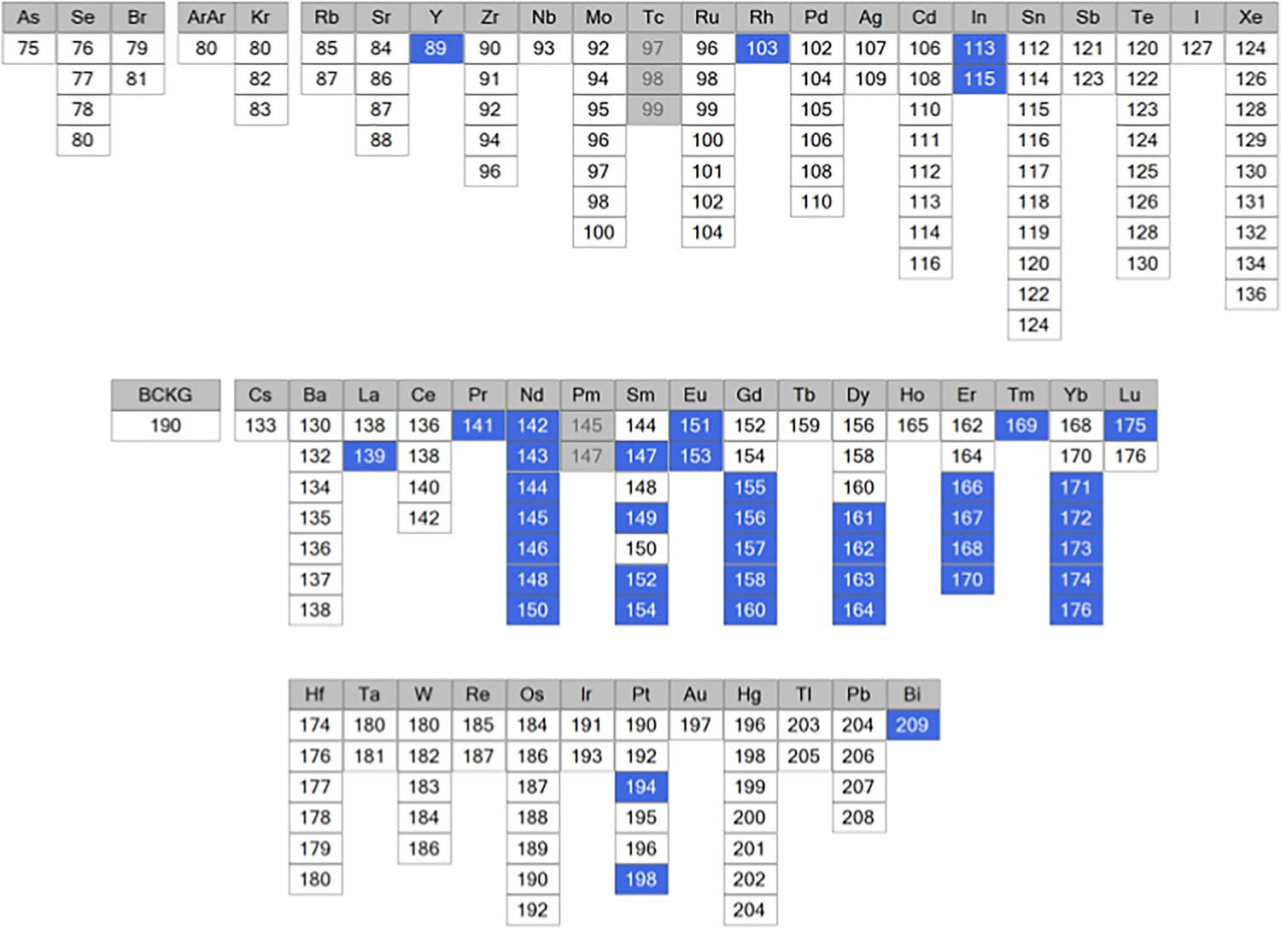
Metals compatible with imaging mass cytometry, recapitulates the 42 metals (in blue) used in published studies using imaging mass cytometry. To note that iridium (191/193) used for nuclei detection is not included and Rh103 is mainly used for counterstaining

Although the majority of metals used for IMC are not naturally present in biological samples, in cancer studies, metal contamination is a risk in samples from patients who were treated with platinum salts (e.g., cisplatin, oxaliplatin) or who underwent imaging using contrast agents with gadolinium that interferes with signal detection. If known, the detection of such therapeutic/diagnostic metals by IMC can be relevant for biodistribution assessment [9, 10]. Moreover, ruthenium can also be used for counterstaining. It binds evenly to cells and when combined with iridium staining mimics hematoxylin and eosin staining [11]. Of note, it is possible to use metal-tagged secondary antibodies to amplify a weak signal or to detect an unconjugated primary antibody not suitable for conjugation. However, such as for IF or IHC, the use of a secondary antibody adds an extra step of staining with the unconjugated antibody first, the secondary antibody, and, at the end, the IMC panel [12]. The use of a secondary antibody might increase the background and has to be tested for each tissue.

### Immunostaining

**Fig. 1:**
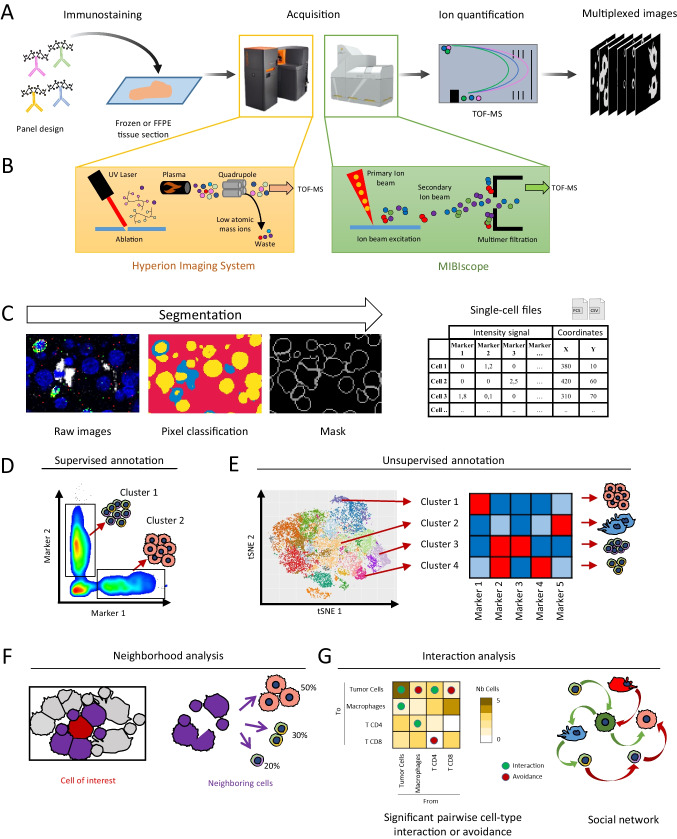
Imaging mass cytometry: principle and applications. (**A**) Imaging mass cytometry workflow. Frozen or FFPE tissues are incubated with metal-conjugated antibodies as done for IHC. Then, slides are inserted into the analyzer (Hyperion Imaging System or MIBIscope) for data acquisition. Metals are ionized and quantified by mass spectrometry by time of flight (TOF–MS). Multiplexed images are reconstituted according the metal abundance per pixel. Hyperion Imaging System and MIBIscope images are generated with the respective software system. (**B**) Hyperion Imaging System (HIS) and MIBIscope acquisition systems. HIS (left panel) uses an UV laser for tissue ablation. Tissue rasterization generates a cloud of biological material that is ionized by inductively coupled plasma. Ions are then filtered by a quadrupole mass spectrometer to discard low atomic mass elements. High mass atomic ions are quantified by TOF–MS. MIBIscope (right panel) uses an O_2_^+^ duoplasmotron primary ion beam to generate secondary ions. Single secondary ions are filtered and quantified by TOF–MS. (**C**) Single-cell file generation. For cell segmentation pixels from TIFF images are classified into nucleus (yellow), cytoplasm (blue), and background (red). All markers can be used for pixel classification. After classification, the cell boundary is determined and a segmentation mask is generated. The combination of single channel images and segmentation mask allows generating a single-cell file suitable for downstream analysis. This file associates, for each cell, its spatial coordinates and the signal intensity of each tested marker. (**D**, **E**) Cell annotation and investigation. From the single-cell files, cell subtypes can be identified. (**D**) The annotation can be done manually, as done during the analysis of a cell suspension by successive gating. (**E**) For cell exploration, unsupervised clustering can be used and its results can be visualized with dimensional reduction tools (e.g., t-SNE, UMAP) in which a color corresponds to a single-cell cluster. Each cluster is identified on the basis of the expression level of each marker visualized by a heatmap. (**F**, **G**) Spatial analysis. (**F**) The spatial coordinates of each cell are used to identify cells in direct contact (purple) with the cell of interest (red). Then, such neighboring cells can be analyzed on their own to determine their composition. (**G**) Cell interactions between all identified clusters can be comprehensively analyzed and visualized in heatmaps. *X*-axis, cell clusters of interest (from); *Y*-axis, cell clusters in contact with the cluster of interest (to); green dots, interactions; red dots, avoidance between clusters. The color intensity indicates the number of cells in contact with the cluster of interest. From the interaction analysis, a cell network for the tissue can be determined

For both the MIBIscope and Hyperion technologies, the staining protocol is based on classical IHC procedures and can be used with snap-frozen, formalin-fixed paraffin-embedded (FFPE) tissue and also plastic samples (Fig. 1(A)) [13, 14]. For FFPE samples, slides must be deparaffinized and the antigen retrieval step done before staining. This means that all antibodies must be validated in the same antigen-retrieval condition (pH, temperature, timing). Each user must find the best antigen retrieval conditions in function of the sample type. Similarly, the staining procedure can be customized and adapted for each antibody and tissue. We identified studies that compared the signal/background ratio of each antibody in function of the staining time and temperature to optimize the staining conditions. These studies give useful information on the best immunodetection conditions, on sequential staining with different antibody panels, and on antibodies/markers that are not compatible with IMC [12, 15].

In addition to antibodies, DNA probes can be conjugated with metal tags to detect mRNAs. This needs to add an in situ hybridization (ISH) step prior the immunohistochemistry. The method, called RNAscope, offers a great opportunity to extend investigations by combining transcriptomic and proteomic approaches. It allows the detection of biomarkers for which no antibody has been developed and validated, poorly expressed, or concentrated such as secreted factors (cytokines, chemokines). This requires to validate the compatibility of each antibody with the ISH procedure. Indeed, not all antigens are conserved after the tissue treatment during ISH [16].

### Differences between the Hyperion Imaging System and MIBIscope

The HIS and MIBIscope use the same antibodies and staining protocols, but MIBIscope requires gold-coated slides. These two approaches rely on different signal acquisition systems. HIS uses a UV laser for tissue ablation. When a pixel is ablated (i.e., a laser shot), a cloud of volatile biological material is sent to the CyTOF, ionized by the plasma, and then ions are quantified by TOF–MS (Fig. 1(B)). HIS uses a quadrupole mass spectrometer to eliminate light elements with an atomic mass < 80 Da before detection. The image resolution corresponds to the laser size (1 µm^2^). The acquisition speed is ~ 60 min/mm^2^ (400 µm^2^/s) with the last generation analyzer (https://www.fluidigm.com/). MIBIscope uses a O_2_^+^ duoplasmotron primary ion beam to liberate secondary ions from the metal elements. Then, the secondary ion cloud is sent to the TOF–MS device for quantification after multimer elimination (Fig. 1(B)). This detection system has three main differences compared with the HIS detection system. First, as the primary ion beam does not destruct the tissue, several acquisition rounds can be performed at different speeds and resolutions. Second, the resolution is adjustable, up to 260 nm. However, increasing the resolution increases the acquisition time. For example, at a resolution of 500 nm, 30 min is needed to rasterize 1mm^2^ of tissue. Third, the TOF–MS device of MIBIscope detects and quantifies all elements, from hydrogen to uranium. This has been used to quantify and use ^12^C and ^31^P, which are naturally present in tissues, as natural counterstain, or to quantify ^56^Fe in FFPE spleen tissue sections for correlating its presence with the amount of heme oxygenase-1 in macrophages [17]. Keren et al. also claimed that MIBIscope is more sensitive than HIS [18], although there is no comparative study published, to our knowledge.

## Image processing and single-cell analysis

High-plex imaging allows visualizing the expression of each marker, alone, or in combination with the other targeted molecules, to obtain a precise picture of the tissue architecture, cell distribution, and biomarker expression. This requires multiple levels of data analysis.

### Cell segmentation

Post-acquisition data processing is required for single-cell analysis. First, images are exported into individual TIFF-OMES files and pre-cleaned (denoising, filtering outlier signals). Then, each cell is identified and individualized. Besides commercial software tools, such as AQUA™ (Navigate BioPharma Inc), Visiopharm®, or HALO® (Indica labs), some open-source software solutions have been developed and are widely used. ImcSegmentationPipeline, the most common, was developed by Bodenmiller’s group and combines multiple steps using different open source software tools [19]. Briefly, cell segmentation relies on pixel classification (nucleus, cytoplasm, background) using Ilastik [20]. Then, a probability map is generated to determine the cell boundaries and to generate a segmentation mask with CellProfiler [21] (Fig. 1(C)). The mask, combined with the individual OMES-TIFF file, is exported as a single-cell file, usually a.fcs or.csv file, that recapitulates the signal intensity and spatial coordinates of each marker in each cell (Fig. 1(C)). These data can be used for cell annotation and in-depth analysis using a flow cytometry software, such as FlowJo, CytoBank [22], OMIQ (https://omiq.ai), or dedicated R packages.

Pixel classification and cell segmentation are the most limiting steps of IMC data analysis. There are many attempts to facilitate and accelerate cell segmentation. such as automated background removal [23], signal normalization and spillover compensation [24], and also automated segmentation tools, such as DeepCell. DeepCell is a data labeling software that is based on the Mesmer deep learning algorithm and that uses TissueNet, a large and comprehensive cell segmentation dataset [25]. However, after cell segmentation, aberrant cell phenotypes are often clustered due to miss-discrimination of the two cell membranes in a high cell density area of the region of interest. For instance, CD19^+^CD3^+^ clusters in lymphoid structures result from the miss-assignment of the CD19 and CD3 signals in areas where CD19^+^CD3^−^ B cells are in close vicinity to CD19^−^CD3^+^ T cells. These clusters can also be analyzed on their own because they may reflect a spatial signature that represents a surrogate of tertiary lymphoid structures. Alternatively, they can be used to determine a segmentation quality index based on aberrant cluster structures. To circumvent this kind of issue and improve cell identification, Bai et al. released a method called REinforcement Dynamic Spillover EliminAtion to reassign pixels near the periphery between adjacent cells. However, it can only correct lateral marker spillover, but not signals due to overlaps [26].

Some studies tried to bypass the segmentation step. For instance, Allam et al. developed a pixel-based analysis method. After raw data cleaning and normalization, they compared clustering at the pixel level and segmentation clustering at the cell level. According to their results, pixel-level clustering seems to provide a better separation between clusters and cell phenotypes with less background noise than the cell-level segmentation method [27].

### Cell annotation

In this step, each cell is annotated on the basis of its phenotype and functional state. Cell annotation can be done manually (Fig. 1(D)) or by unsupervised clustering for cell exploration (e.g., Phenograph or FlowSOM). Unsupervised methods allow the unbiased cell identification and, potentially, the discovery of unanticipated cell phenotypes. After annotation, the expression of functional markers can be quantified to evaluate cell functionality or maturation, and the cell density and frequency can be calculated. The results can be visualized using uniform manifold approximation and projection [28] or t-distributed stochastic neighbor embedding [29]. To determine the signature of a specific cell subgroup, single-cell files can be processed with predictive algorithms, such as CITRUS that combines unsupervised clustering and signature identification (Fig. 1(E)) [30].

### Spatial distribution and interactions

Once a cell population of interest (POI) has been determined, the neighborhood analysis can be performed. The data of cells in contact with a specific POI can be exported in a single file for individual analysis of neighbor cell composition to identify preferred partnerships (Fig. 1(F)). Spatial features can be identified by pairwise enrichment to determine significant enrichments or avoidances between cell clusters. By adjusting the distance between cells, it allows defining spatial signatures that involve two or more cell clusters, and to determine a cell community/network (Fig. 1(G)) [31]. Some open-source software tools are available to determine the cell spatial distribution, such as histoCAT and ImaCYTE [32, 33]. These tools integrate many plugins for single-cell analysis, such as dot-plots, dimensional reduction, and clustering. Of note, R packages also have been developed. They require bioinformatics skills, but they are more flexible than software tools and may be more adapted for such analysis. ImcRtools and cytomapper are two R Bioconductor packages developed by Bodenmiller’s group for IMC image processing and analysis [29, 30]. Data processing and analysis are still complicated (for many scientists with limited bioinformatic skills) and time-consuming, but they allow fully exploiting IMC data for quantitative tissue profiling. The diversity of analysis pipelines widens the questions that can be addressed using IMC.

### 3D modeling and high-resolution imaging

3D imaging offers new perspectives for tissue analysis. As a proof of concept, Kuett et al. analyzed IMC 152 serial tumor tissue sections. After cell segmentation, they reconstituted the tumor in 3D for spatial analysis. They compared the proximity between cell clusters and blood vessels using 2D and 3D data and found that the 2D approach significantly overestimated this distance [34]. Rovira-Clavé et al. studied cisplatin distribution in cultured cells by IMC. For this purpose, they replaced the oxygen duoplasmatron source of a MIBIscope by a cesium primary beam. This modification requires the use of halogen-loaded single-strand DNA-conjugated antibodies instead of lanthanide-conjugated antibodies, but allows a resolution of ~ 30 nm (compared with the HIS and MIBIscope resolutions of 1 µm and 260 nm, respectively). For the first time, they could detect cisplatin at the subcellular level and could correlate cisplatin localization with five subnuclear structures in function of the cell type (e.g., resistant to treatment or not) and treatment combination [9].

## IMC in preclinical research

Preclinical models, which are becoming more and more sophisticated, may allow unraveling complex biological mechanisms and IMC may improve their understanding. To date, few in vivo preclinical studies and even fewer in vitro studies based on IMC have been published.

As MIBIscope and HIS can detect platinum isotopes, they have been used to monitor platinum-based chemotherapy bio-distribution in vivo. Chang et al. assessed platinum bio-distribution and clearance in a patient-derived xenograft model of pancreatic cancer. Using a 14-antibody panel, they found that platinum binds to collagen fibers in tumor and normal tissues, but with different persistence [10]. Melin et al. monitored the radiation side effects in liver in mice by assessing the spatiotemporal alterations of the liver immune contexture, using a 20-antibody panel. They observed that the proportion of neutrophils, macrophages, and T cells increased and that these cells preferentially clustered near the central veins after irradiation. Moreover collagen accumulated, indicating pericentral fibrosis [35]. Zabransky et al. compared the TME, notably the immune infiltrate, in four different syngeneic hepatocellular carcinoma (HCC) mouse models treated with anti-PD-1 antibodies. In three models, they found immune profiles that were similar to those observed in human HCC. IMC data analysis showed that the accumulation of M2-like tumor-associated macrophages and the strong interaction between CAFs and T cells were linked to treatment resistance [36]. In the mT3 KPC mouse model of pancreatic cancer, Peran et al. simultaneously detected RNAs and proteins by IMC to investigate CAF interactions within the TME. They found that cadherin 11 (CDH11) expression was associated with CAF pro-tumor activity, which could be inhibited by an anti-CDH11 antibody. Deep immunophenotyping showed that anti-CDH11 antibodies decreased the frequency of FOXP3^+^ T cells in the tumor [37]. To decipher the lung TME and the effect of the KRAS^G12C^ inhibitor MRTX-1257, Van Maldegem et al. set up a 27-antibody panel for frozen tissue analysis and developed their own segmentation pipeline named *imcyto*. Single-cell IMC analysis permitted a thorough description of the immune landscape of KRAS-mutated lung tumors, highlighted the strong PD-L1 expression in macrophages, and the preferential localization of PD-L1-positive macrophages next to dendritic cells, which potentially counteracted their activation and consequently T cell activation through the immunosuppressive PD-1/PD-L1 pathway. Interestingly, upon KRAS^G12C^ inhibition, the authors observed that macrophage-dendritic cell interactions disappeared [38].

IMC can also be very useful to compare different drug delivery methods and their pharmacodynamics. Liu et al. used IMC to evaluate the benefit of nanofluidic drug-eluting seeds (NDES) for intratumoral delivery of anti-PD-L1 and anti-CD40 antibodies in a triple negative breast cancer mouse model. They demonstrated that NDES improves immunotherapy efficacy and promotes abscopal effects compared with intraperitoneal injection. To obtain these results, they used a 15-antibody panel that allowed identifying and localizing 21 immune cell populations. They observed that NDES efficacy was associated with higher tumor infiltration by CD4^+^ and CD8^+^ T cells compared with drug delivery by intraperitoneal injection. The neighborhood analysis showed increased interactions between cytotoxic T cells, dendritic cells, and cancer cells in the NDES group [39]. IMC has also been used to follow nanoparticle-mediated drug delivery into the tumor mass. Strittmatter et al. combined mass spectrometry imaging and IMC in patient-derived xenograft mouse models of lung, colon, and ovarian cancer to follow the biodistribution and the effect on the TME of encapsulated AZD2811, an aurora kinase B inhibitor. They designed an IMC 27-antibody panel to assess the TME cell composition (e.g., immune, stromal, muscle, endothelial, epithelial cells) and the cell metabolic and functional status (hypoxia, proliferation, exhaustion, M2 balance, epithelial-mesenchymal transition, and glucose metabolism). Then, using MSI, they precisely quantified and analyzed the spatial distribution of AZD2811-loaded nanoparticles on the same tissue section. Thus, the multimodal approach revealed that NP encapsulated AZD2811 was preferentially distributed in regions rich in macrophages. Although still preliminary, this work shows the benefits of combining two imaging approaches to better understand the nanomedicine-delivered drug distribution and their mechanisms of action within the tumor [40]. In another study, Strittmatter et al. combined again mass spectrometry imaging and IMC to evaluate the intratumoral distribution and the effect of gemcitabine and its phosphorylated metabolites in the KPC mouse model of pancreatic cancer. They used a 25-antibody panel to discriminate the effects of the drug and its metabolites on the tumor and immune cell state (metabolism, signaling, DNA damage, proliferation, mitosis). They found that gemcitabine metabolites induced DNA damage in highly proliferative areas. This work provided evidences that IMC and mass spectrometry imaging-based multimodal molecular imaging is a powerful combination to evaluate nanoparticle delivery, to localize and quantify metabolites with MSI and to study the tumor microenvironment modification of such treatment with IMC [41].

In addition, IMC is a comprehensive approach to study small structures, such as spheroids and organoids. For instance, Lotsberg et al. set up a spheroid culture system that mimics cancer-stromal cell interactions in non-small cell lung cancer to study how these interactions influence epithelial-to-mesenchymal transition and treatment resistance. They co-cultured EGFR inhibitor-resistant lung cancer clones with fibroblasts and investigated how co-culturing modifies the spheroid cell composition using a 19-antibody panel to assess phenotypic and proliferation markers and to quantify each cell type and also cell–cell interactions within the spheroids. The single-cell analysis revealed a strong tumor cell heterogeneity with an inverse association between mesenchymal cell marker expression (associated with resistance) and the capacity to form compact spheroids. Moreover, they observed different stromal cell interactions in function of the cancer cell phenotype [42].

## IMC in clinical research

Spatial single-cell analysis can be used to comprehensively monitor the immune contexture in situ and to follow and localize the expression of biomarkers in order to identify signatures, including cell–cell interactions. In this section, we describe how IMC may contribute to better characterize solid tumors to discover and decipher new complex biological mechanisms for patient stratification in view of precision medicine.

### Identification of cells with complex phenotypes

By analyzing skin biopsies from patients with mycosis fungoides, a common form of cutaneous T cell lymphoma, Guo et al. identified by mass cytometry (CyTOF) cells that express CD25, CD45RO, and CD27 and that they considered to be Treg-like cells. Then, they used a 36-antibody panel to confirm by IMC their presence within the tumor and to describe specific cell interaction networks and the expression of biomarkers associated with the disease stage. The combination of CyTOF and IMC also highlighted the high inter-patient heterogeneity [43]. Similarly, Wang et al., who previously described significant modifications of the immune contexture by comparing healthy dura mater and primary meningioma samples by single-cell RNA sequencing, used IMC to assess the TME in meningioma. Using a 20-antibody panel to identify and localize immune cell-stroma cell interactions, they found that the CD8^+^ T cell composition was modified with a preponderance of resident memory T cells within the tumor, compared with the naive/central memory balance of CD8^+^ T cells in healthy dura mater samples. They then characterized macrophage distribution, and identified border-associated macrophages and suggested spatial relationships with the co-localized T cells and antigen-presenting cells [44]. Similarly, Sanmamed et al. identified by CyTOF a dysfunctional subpopulation of CD8^+^ T cells that accumulates in the TME and that is associated with poor response to immune checkpoint inhibitors (ICIs) in advanced non-small-cell lung cancer. They found that these cells strongly proliferate, but produce little or no interferon gamma (IFNγ). Then, with a 35-antibody panel, they showed that this proliferative T cell population (Ki-67^+^ CD28^+^) expresses many immune checkpoint molecules (PD-1^+^ LAG-3^+^ TIM-3^+^) and is terminally differentiated (EOMES^high^ TBET^low^) [45].

### Spatial tumor heterogeneity and discovery of unexpected cell phenotypes

Besides their spatial distribution and interactions, IMC allows assessing the tumor cell heterogeneity at the single-cell level. Bodenmiller’s group, a pioneer in this field, analyzed 352 breast cancer samples. Based on the cell phenotype and interaction analyses, they identified 23 individual cell communities with a 35-antibody panel and defined 18 novel breast cancer subgroups [31]. Similarly, Sheng et al. quantified 36 biomarkers in 134 HCC samples and identified 16 cell communities based on the interactions of normal and tumoral hepatocytes, and endothelial and immune cells [46].

Furthermore, cell heterogeneity assessment by IMC may lead to the identification of unanticipated cell phenotypes or functions. For instance, Ferrian et al. published a case report about a patient with endometrial and colorectal carcinoma who received chemotherapy and brachytherapy as first-line therapy before anti-PD-1 antibodies (nivolumab) as second-line therapy. The patient responded well to these treatments, but presented severe side effects, particularly gastritis. In situ hybridization analysis showed high IFNγ expression in cancer samples and IMC analysis of gastric biopsies revealed a strong immune cell infiltration (> 50%) suggesting that the main source of IFNγ was the immune infiltrate. Unexpectedly, an unbiaised analysis showed that IFNγ was predominantly secreted by a specific and unknown cluster of epithelial cells (PanCK^+^ HLA-DR^+^ Ki-67^+^ IFNγ^+^) instead of the immune cells [47]. Zheng et al. analyzed by CyTOF the immune infiltrate in lung cancer. Unsupervised analysis clustered a subpopulation of PD-L1-expressing CD8^+^ T cells while PD-L1 I is mainly expressed by tumor and myeloid cells. Using an 18-antibody panel they studied T cell distribution in cancer tissue. Consistently with the CyTOF data, by unsupervised clustering they identified 11 distinct CD8^+^ T cell clusters, including the one expressing PD-L1. By neighborhood analysis, they showed that the PD-L1^+^CD8^+^ T cell cluster was preferentially in contact with PD-1^+^CD38^+^CD8^+^ T cells, suggesting that such interaction contributes to inhibit the immune response and promotes immune escape. Lastly, they demonstrated, in vitro, that PD-L1-expressing CD8^+^ T cells suppress effector T cell functions [48].

### Beyond phenotypic marker detection

The published application examples demonstrate the high diversity of questions that can be addressed with IMC. For instance, antibodies against phosphokinases can be added in the panel to assess also cell signaling. IMC panels may include antibodies against enzymes involved in metabolic pathways, bacterial markers, or DNA probes to detect mRNA transcripts. Guo et al. compared the phosphorylation state of the translational inhibitor eIF4E in tumor cells from patients with postpartum breast cancer by IMC. They found that that postpartum breast cancer samples contained cancer cells that strongly expressed phosphorylated eIF4E (active form) and that tumor CD8^+^ T cells displayed markers of dysfunction. Based on these results, the authors suggested that blocking phosphorylated eI4E might enhance the efficacy of immunotherapies [49]. Feng et al. used IMC with a 14-antibody panel that included also antibodies against lipoteichoic acid and lipopolysaccharides to target Gram^+^ and Gram^−^ bacteria, respectively. They could correlate the presence of bacteria in breast cancer samples with cancer subtypes and immune infiltrate [50]. Using the MIBI technology, Hartmann et al. combined immune and metabolic markers in a 36-antibody panel. They compared the metabolic profile of T cells and epithelial cells from colorectal carcinoma tissues. They identified two metabolic profiles of CD8^+^ cells that express CD39 and PD-1. This suggests two functional states that allow differentiating between exhausted and activated cells [51]. The concomitant detection of proteins and mRNAs represents a great opportunity to better describe a phenotype and to detect new targets for which no efficient antibody has been validated yet [16]. For instance, Hoch et al. studied chemokine RNA and immune cell marker protein expression by IMC in metastatic melanoma samples and could detect *CXCL9* and *CXCL10* expression in dysfunctional T cell patches that express *CXCL13* [52].

### Deciphering complex biological mechanisms

To investigate the immune contribution of the COVID-19 mRNA-1273 vaccine to the regression of metastatic salivary gland myoepithelial carcinoma, Sousa et al. used IMC to study pre- and post- vaccination metastatic and primary tumor biopsies. They observed a complete change in the immune balance, from higher frequency of M2 macrophages and neutrophils in pre-vaccination samples to preponderance of lymphocytes (T, B, and natural killer cells) after vaccination [53]. Alnajar et al. explored the TME of a metastatic sarcomatoid urothelial carcinoma in a patient presenting prolonged response to pembrolizumab (anti-PD-1 antibody) after primary resistance to chemotherapy. Next-generation sequencing revealed that the tumor mutational burden was decreased accompanied by *PD-L1* genomic amplification in cancer cells that was confirmed by IHC. Moreover, IMC showed that PD-L1 was mainly secreted by tumor cells and not myeloid cells [54].

### Patient stratification

Processing IMC data from a large number of patients may lead to the tumor re-classification for patient stratification and prognosis. For instance, besides revealing the tumor cell heterogeneity and immune contexture, Jackson et al. characterized the tissue architecture and identified specific cellular features that allowed determining 18 novel breast cancer subgroups that are associated with distinct clinical outcomes [31]. Danemberg et al. extended this work to 693 breast tumor samples and integrated IMC data with clinical and genomic data. They showed that the proportions of granulocytes, antigen-presenting cells, vasculature, and T cells with suppressive phenotype in the TME vary in function of their localization, thus forming distinct structures that may predict survival and improve patient stratification [55]. Feng et al. identified in muscle invasive bladder cancer three major spatial distribution patterns of the epithelial cell markers pan-cytokeratin and E-cadherin. These three cancer types presented different immune cell profiles (enrichment/composition) that may influence the tumor course and response to treatment [56].

### Pathology comparison

IMC is also a useful approach for group comparison. It generates objective data (marker expression, cell phenotype abundance, and cell interaction scores) that allow comparing the clinical status in different cancer types or cohorts. Colombo et al. explored the immune landscape in diffuse large B cell lymphoma (DLBCL) samples with a 32-antibody panel to compare their immune contexture with that of Hodgkin’s lymphoma, which is sensitive to ICI, in order to understand DLBCL poor response to ICI. IMC revealed an association between CXCR3 expression and T cell infiltration in immune desert regions of DLBCL samples [57]. To understand how obesity influences breast cancer course, McDowel et al. used a 35-antibody panel to objectively compare the TME of breast cancer metastases from patients with high (obesity) and normal body mass index. IMC showed that neutrophils and proliferating (Ki67^+^) tumor cells were increased in patients with obesity. The authors suggested that due to neutrophil oxidative stress, vascular permeability was modified and promoted the migration of neutrophils and tumor cells [58]. Risom et al. monitored the progression of pre-invasive breast cancer lesions (i.e., ductal carcinoma in situ (DCIS)) into invasive breast cancer using a 37-antibody panel to extract 433 parameters in tumor samples from matched patients with DCIS, invasive breast cancer (progressors), and DCIS that did not progress (non-progressors). They found that the location/function of stromal cells (particularly CAFs) and myoepithelium, rather than cancer cells, constituted a prognostic feature of DCIS-to-invasive breast cancer transition [59].

### Treatment response prediction

The identification of predictive markers is a current challenge in oncology. Martinez-Morilla et al. assessed the expression of different biomarkers by IMC and associated β-2 microglobulin expression with a better response to immunotherapy in melanoma [60]. Abdulrahman et al. combined single-cell RNA sequencing for TCR repertoire profiling and bulk sequencing to characterize the transcriptional states of clonally expanded tumor-infiltrating T cells in oropharyngeal squamous cell carcinoma samples. They identified two groups based on their immune status: immune responsive (IR +) and non-responsive (IR −). They observed that survival was better in the IR + group than in IR − patients. Then, using IMC and a 33-antibody panel, they identified 51 cell clusters (including 30 immune cell clusters and 14 tumor cell clusters) and specific spatial features that characterize IR + tumors (co-localization of resident CD8^+^ T cells and DC) and IR − tumors (interactions between lymphocytes and immunosuppressive myeloid cells) [61].

### Improving cancer therapies

To improve treatment efficacy, an important challenge is to understand how treatments influence the tumor ecosystem. Ho et al. evaluated the benefit of neoadjuvant cabozantinib and nivolumab in patients with HCC. At resection time, they compared responders and non-responders by IMC. In responders, their TME was enriched in effector T cells, and B cells were spatially rearranged. Conversely, in non-responders, the proportion of CD163^+^ARG1^+^ macrophages was increased in the TME, and this may partly explain the resistance to ICI [62]. Fonkoua et al. studied by IMC TME changes in patients with metastatic gastroesophageal adenocarcinoma after ICI followed by ramucirumab/paclitaxel compared with pre-ICI samples. They found that the sequential combination prevented Treg infiltration and preserved the pool of cytotoxic T cells [63]. Yang et al. observed similar TME modifications in six patients with advanced rectal cancer treated with neoadjuvant chemoradiotherapy by comparing the TME by IMC of patients with and without complete pathological response. They showed that complete response (*n* = 3) was associated with an increase in the proportion of cytotoxic lymphocytes and a decrease in tumor-associated M2 macrophages and Treg cells [64]. Moldoveanu et al. mapped by IMC the tumor immune compartment of melanoma samples after ICI. They reported that proximity of antigen-experienced cytotoxic T cells with tumor cells and a high proportion of proliferating experienced CD8^+^ T cells were associated with positive response to ICI [65].

## Conclusion

In this review, we described the potential contributions of IMC in the field of immuno-oncology. From the study of biomarker expression and distribution to the identification of a predictive signature based on cell–cell interactions, IMC allows an in-depth and comprehensive study of the tumor ecosystem. The continuous release of new computational tools and methods for multiplexed image and single-cell analysis confirms the constant progress of this technology.

The importance of the immune context in the response to treatment is now acknowledged. The emergence of the immunoscore to predict the response to treatment in colorectal cancer has clearly demonstrated the importance of studying the immune status of patients before treatment [66, 67]. Therefore, IMC, with its spatial approach, can play a crucial role in the discovery of predictive signatures by comparing the TME before and after treatment and between responders and non-responders. Altogether, IMC-based studies will significantly contribute to the discovery of predictive signatures and mechanisms of resistance, and consequently also to the development of new therapeutic strategies for precision medicine.
